# School climate and creativity as predictors of psychological resilience among college students

**DOI:** 10.3389/fpsyt.2025.1576023

**Published:** 2025-10-29

**Authors:** Yu Yan, RongHua Li

**Affiliations:** ^1^ College of Resource Environment and Tourism, Capital Normal University, Beijing, China; ^2^ Higher Education Research Institute, Shantou University, Shantou, Guangdong, China

**Keywords:** psychological resilience, decision tree, prediction, creativity, school climate

## Abstract

**Background:**

The overuse of smartphones prevails in college that strongly links to poor school climate, and brings great academic and psychological challenges to college students despite promotion in convenience of life and study. While moderate negative emotions caused by school climate can enhance creativity and further foster psychological resilience, severe adverse effects would impede development of resilience.

**Methods:**

This study aims to examine the prediction of four factors (school climate, creativity, social anxiety and sense of place) on psychological resilience by developing a prediction model based on the decision tree algorithm. The analysis was conducted in Modeler 18.0 with C5.0 algorithm, and the model accuracy is 78.26%. A sample of 607 college students participated, completing five established scales: Connor-Davidson Resilience Scale, Runco Ideational Behavior Scale, Perceived School Climate Scale, Social Anxiety Subscale of the Self-Consciousness Scale and Sense of Place Scale.

**Results:**

Psychological resilience was predicted by four factors, in order of significance: creativity, school climate, social anxiety and sense of place. Additionally, creativity emerged as the most significant predictor of psychological resilience with a notable margin over the other three factors.

**Implications:**

This study offers valuable insights for researchers to understand predictive relationships and capabilities of creativity and school climate on psychological resilience, provides a tool for school to identify, anticipate and decrease students’ psychological crisis, and further contributes to designing targeted development and management programs that enhance mental health support for students.

## Introduction

In the digital era, the proliferation of electronic information technologies has driven an exponential increase in global smartphone adoption, with students constituting the largest user segment. The 44th Statistical Report on China’s Internet Development, released by the China Internet Network Information Center has stated that the number of smartphone users in China had reached 1.096 billion, and 99.7% of internet users accessed the web via smartphones by June 2024, with university students representing the predominant demographic (1). Adolescents and university students are widely recognized as a high-risk population for developing problematic smartphone use (PSU) and excessive engagement with social media. A growing body of research has documented associations between PSU and a spectrum of adverse psychological outcomes, including elevated levels of depression, anxiety, and perceived stress, all of which may undermine students’ psychological resilience—defined as the capacity to cope with stress and adapt effectively in the face of adversity, and considered a key target for interventions addressing depression, anxiety, and stress (2). Nevertheless, research remains limited on the factors that influence the development of psychological resilience among adolescents and young adults in the context of pervasive smartphone use.

Given that adolescents spend a substantial proportion of their time in educational settings, the school climate constitutes a critical developmental milieu in which behavioral patterns, social relationships, and psychological resources are shaped and reinforced. Empirical studies have reported a negative association between school climate and problematic smartphone and social media use (3). In addition, hierarchical regression analyses have demonstrated that smartphone addiction negatively affects students’ creativity while simultaneously exerting a positive effect on negative affect (4, 5). It is also noteworthy that PSU has been consistently linked to elevated levels of social anxiety, with a growing body of research reporting a robust positive association between smartphone addiction and heightened social anxiety (6, 7). Taken together, these findings suggest that excessive smartphone use among university students is closely related to school climate, creativity, and social anxiety. Nevertheless, in the context of pervasive smartphone use, the prediction of students’ psychological resilience on these key psychosocial variables remains insufficiently understood and warrants further empirical investigation.

Therefore, this study aims to empirically investigate the prediction model of psychological resilience on four critical determinants—school climate, creativity, social anxiety, and sense of place—among university students in the context of pervasive smartphone use. By clarifying these relationships, the study aims to provide empirical evidence to guide the development of targeted interventions and evidence-based strategies for fostering mental health and adaptive functioning among university students. It is worth noting that PSU should be regarded as a significant contextual background shaping the psychological landscape of Chinese university students in this study, rather than being treated as an independent research variable.

## Theory and related studies

### Psychological resilience

Psychological resilience is a multifaceted construct with significant implications for health sciences. The definition of psychological resilience evolved over the time, assuming different shades of meaning (8). Initially, resilience was defined as the ability to successfully adjust to adverse or threatening situations and to withstand trauma (9, 10). Later, psychological resilience was thought to be a dynamic process that allows people find a new balance in adversity and turn it into positive opportunity for personal growth (11). In this process, individuals acquire new competencies and a revitalized sense of personal effectiveness and self-growth. In broader discussions, some scholars have conceptualized resilience as a stable personality trait (12, 13), aligned with the ‘ego resiliency’ theory (14), which claims that protective factors against stress are primarily provided by inherent personality characteristics. Alternatively, other scholars have defined resilience as a process (15, 16), emphasizing the dynamic interaction between individuals and their environment, where both protective and risk factors operate concurrently, with the final outcome resulting from their interaction. Richardson et al. (17, 18 sought to combine two kinds of views by proposing that psychological resilience is shaped by both genetic factors and personal experience, and stated that it is not only an innate propensity but also provide opportunity for self-reflection and self-redefinition. Based on comprehensive literature review and analysis, Sisto et al. (8) put forward a compound definition for psychological resilience, encompassing five elements: capacity for recovery, functional characteristics of the individual, resilience to rebound, dynamic and evolving process, adaptive response to life circumstances. This definition contributes to the conceptual unification of resilience.

Psychological resilience serves as a measure of the ability to cope with stress in face of adversity and the key target for addressing issues related to depression, anxiety, and stress (13). It plays an important role in university life where students must navigate adaptation process to constant changes. When students encounter distress such as environmental discomfort, academic pressure, social difficulties or emotional problems, their psychosocial and emotional development and mental health may suffer if they fail to balance internal strengths and potential challenges (19). Conversely, the promotion of resilience can have positive effects on buffering and reducing stress-related negative emotions (20), improve students’ academic performance (21) and life satisfaction (22), and better equip them to manage adverse events and dilemma (23).

In order to quantitatively measure resilience level of individuals, a mass of assessment tools was produced. Satapathy et al. (24) utilized 12 resilience-focused scales, each with distinct features (e.g., targeted age group, number of items, scale type). Specifically, CD-RISC stands out as one of the most broadly applied resilience measures and has been validated in clinical settings (13, 25). While most scales are primarily designed for screening purposes, some partial scales can also be employed for profiling and intervention (24).

The prediction of psychological resilience has been explored in limited studies. Bonanno et al. (26) identified demographic factors (e.g., gender, age, race/ethnicity, education), resources (e.g., income changes, social support), and life stressors as key predictors based on multivariate analyses. Besides, early childhood characteristics especially father-child relationship was noted as predictors for psychological resilience (27). Biophysiological features such as elevated vagal activity during stress anticipation and the ability to restore cortisol-DHEA balance after exposure to stress also proved to be effective in promotion of predictive accuracy (28). However, the predictive power of individuals’ mental characteristics towards resilience remains obscure although correlations between those variables were attentively investigated. Predicting students’ psychological resilience can offer guidance for educators, aiding in mental health assessment, crisis intervention, and fostering persistence toward personal goals (8). Hence, our study centered on mental traits as predictors of resilience holds critical importance.

### Creativity

Creativity is defined as the cognitive ability to produce novel thoughts (29). In 4C model proposed by Kaufman and Beghetto (30), “Big C”, “Pro C”, “Little C” and “Mini C” signified innovation across different domains and magnitudes. Runco (31) considered “Little C” creativity as the ability to solve problem, navigate shifts in society and routine life and generate personal opportunities. Moderate negative emotions brought by the smartphone addiction may facilitate creativity and resilience. Studies have demonstrated a significant positive correlation between psychological resilience and creativity (32, 33). Additionally, Li et al. (34) found that nursing undergraduates with higher creativity exhibited greater psychological resilience, which can be explained by Sheng (35)’s findings that maladjustment negatively associates with creativity, illustrating highly creative individuals are more adaptive and resilient. Based on this evidence, we posited that creativity serves as a predictor of psychological resilience (H1).

### School climate

School climate describes the quality and character of school life as perceived and experienced by individuals (36), which has been primarily examined through three components: teacher-student support, student-student support, and opportunities for autonomy in the classroom. Teacher-student support combines emotional and academic assistance from teachers, while student-student support reflects perceived emotional and learning support among peers. Opportunities for autonomy in the classroom signify sufficient freedom to make choices and decisions during class activities or study (37). Smartphone addiction generally corresponds to poor school climate with weak peer and teacher-student support. The dimensional structure of school climate has developed with many scholars categorizing it into six constructs: teacher support, peer connectedness, school connectedness, affirming diversity, rule clarity and reporting and seeking help (38–40). Even if the correlativity between each construct of school climate and psychological resilience was found varied (41–43), as a whole, school climate was verified positively related to psychological resilience that positive school climate was linked to and favored positive adjustment outcomes (44, 45). We hypothesized perception of school climate as the predictor for psychological resilience (H2).

### Social anxiety

Social anxiety is a dominant and persistent anxiety (46) that consists of three components: (a) distressful, discomfortable and anxious feelings when interacting with others; (b) conscious avoidance of social engagements and (c) anxiety about being judged unfavorably by others (47, 48). Over-reliance on mobile phones has negative impacts on social skills that may further hinder the development of resilience. The association between social anxiety and psychological resilience was documented in several studies (49–53). Findings indicate a significant negative correlation, suggesting that individuals with higher resilience are more at ease in social contexts, whereas enduring or intensifying social anxiety is linked to lower levels of resilience. Thus, we assumed that social anxiety predicts psychological resilience (H3).

### Sense of place

The concept of sense of place was first introduced by Lowenthal (54) and Tuan (55), who described it as both the inherent trait of place and the bond between people and their environment. The connection could be divided into cognitive, behavioral and emotional dimensions whereas emotional perspective was predominant (56). Despite the lack of a universally accepted definition, scholars shared a belief that sense of place arises from the interplay between the environment and individual perception, reflecting the outcomes of human-place interactions (57, 58). Massey argued that sense of place is not static but develops over time (as cited in 59). Jorgensen and Stedman (60) further refined the concept, dividing it into three dimensions (place identity, place attachment and place dependence), as the most influential and widely accepted definition. Being addicted to mobile phones often diminishes people’s connection to their surroundings and thereby brings down their sense of place. Correlation analyses have revealed a positive association between sense of place and psychological resilience (59), which leads to the assumption that sense of place is the predictor for psychological resilience (H4).

## Methods

### Participants

The present study was conducted at a college in Sichuan Province, accommodating more than 17,000 students. Before finalizing the study design, an exploratory focus interview was held with five volunteer respondents to identify the possible predictors of psychological resilience. Creativity, school climate, sense of place and social anxiety were estimated as predictive factors. The survey materials and design were submitted to the Ethics Review Committee of Chengdu Normal University and the principals of sample schools. Following a review of the ethical considerations and research requirements, consent was obtained, and the survey was administered to the students. Prior to participation, all students provided electronic informed consent. The inclusion criteria were threefold: (1) full-time undergraduate students currently enrolled in the college, (2) aged between 18 and 25 years, and (3) having regular smartphone use. Exclusion criteria were: (1) students with self-reported severe psychological disorders or cognitive impairments, and (2) incomplete questionnaire responses. Using a convenient sampling method, a total of 607 voluntary participants filled out the questionnaire. Among them, 304 were sophomores and 303 were juniors. After data collection, we examined the validity of the questionnaires and found out 607 reliable questionnaires. Among the sample group, 155 were male (25.5%) and 452 were female (74.5%), reflecting the predominance of female students in Chinese normal universities, which resulted in a higher proportion of female respondents. In terms of geographic background, 434 students (71.5%) were from rural areas, while 173 (28.5%) were from urban areas, consistent with the demographic composition of college students in the western region of China. Participants’ ages ranged primarily from 18 to 23, with a small minority older than 23. Specifically, 22 participants (3.6%) were 18 years old, 143 (23.6%) were 19, 211 (34.8%) were 20, 159 (26.2%) were 21, 46 (7.6%) were 22, and 23 (3.8%) were above 23.

### Data collection and instruments

The research discussed in this paper adopted the relevant design scheme, and collected the data through online questionnaire survey. The questionnaires were filled out between September 10 and September 15, 2023. As a learning task, the class advisor showed students the QR code of the questionnaire in class meeting that was a scannable bar code with extensive information. Students only need to use electronic devices (e.g., phone, ipad) to scan QR code linked to specific interface for questionnaire fulfillment. In China, QR codes are commonly employed for accessing specific platforms and performing multiple functions, including financial transactions, identity verification and information inquiry. Before students scanned the code, class advisor had introduced the purpose of our research in detail to confirm students’ voluntary participation in survey, which was essential to maintain the research’s impartiality and validity. Consequently, the data collection process in this study carefully respected and safeguarded participants’ autonomy and informed consent.

### Measures

Our questionnaire consisted of six sections: demographic information, Connor-Davidson Resilience Scale, Runco Ideational Behavior Scale, Perceived School Climate Scale, Social Anxiety Subscale of the Self-Consciousness Scale and Sense of Place Scale. Demographic information comprised of sex, age and origin place of student. Five scales were utilized to measure students’ level of psychological resilience, creativity, perceived school climate, social anxiety and sense of place, complied in English initially and subsequently translated into Chinese for this study. We adopted Back-Translation proposed by Brislin (61) to ensure the quality of translation. In this process, researcher A and researcher B complete Chinese-English translation and English-Chinese translation respectively, and researcher C compared the original text, the Chinese translation, and the back-translated English version to evaluate the translation’s accuracy. Before finalizing the questionnaire, we revised and optimized the translation text to guarantee the equivalence of the scale. The scale has been validated for use with Chinese student populations, demonstrating good reliability and validity (e.g., 2, 62). A higher total score indicates a greater level of psychological resilience, school climate, creativity, social anxiety and sense of place. The scale contains no reverse-scored items. A detailed description of each scale is provided below.

### Psychological resilience scale

Participants’ psychological resilience was assessed by 10-item Connor-Davidson Resilience Scale (CD-RISC-10 for short), a condensed version of the original 25-item scale designed to measure an individual’s recoverability from adversity and adapt flexibly to fluctuating external circumstances. Connor and Davidson (13) divided it into five dimensions: ability (e.g., “I adapt well when faced with change”), tolerance for negative emotions (e.g., “I approach difficulties with a sense of humor”), acceptance for change (e.g., “I become stronger through the accumulation of experience”), control (e.g., “I can manage negative emotions, including anger”) and mental influence (e.g., “I can concentrate on my thoughts under pressure”). Campbell-Sills and Stein (63) simplified the scale into a 10-item version. The participants’ feelings, reactions and recognitions were evaluated by 5-point scale, ranging from 1 (completely inconsistent) to 5 (completely consistent). In this study, the scale demonstrated high internal reliability, with a Cronbach’s alpha coefficient of 0.938.

### Innovative behavior scale

This study adopted Innovative Behavior Scale proposed by Janssen (64) through integrating concepts from many scholars. While certain terms were modified to conform to the linguistic habits and lived experiences of college students, the scale still contains 9 items and three dimensions: the generation of innovative ideas (e.g., “I often come up with new ideas when faced with challenges”), the promotion of innovative ideas (e.g., “I will look for managerial support for inventive ideas”), and the realization of innovative idea (e.g., “I will execute creative ideas in real-world applications”). The scale rated on five points reflects individuals’ innovation level, ranging from 1 (strongly object) to 5 (strongly agree). The Cronbach’s alpha coefficient for this scale was 0.917.

### Perceived school climate scale

A 25-item Perceived School Climate Scale (37) was utilized in this study to indicate significant psychological and behavioral effects of perceived or experienced school atmosphere on its members. The scale evaluates three dimensions of school climate: teacher support (the emotional and academic assistance provided by teachers), peer support (emotional and academic support exchanged among students), opportunities for autonomy in the classroom (freedom to make choices or decisions in class or study activities). All items are rated on a 4-point response scale (1 = never, 2 = sometimes; 3 = often; 4 = always). The Cronbach’s alpha coefficient for this scale was 0.888.

### Social anxiety subscale of the self-consciousness scale

The Social Anxiety Subscale of the Self-Consciousness Scale (SASS-CS for short) used in this study was proposed by Fenigstein et al. (65), and then some wording was revised more easily to understand by Scheier and Carver (66). The SASS-CS is applied to measure the subjective experience of social anxiety in social contexts, and consists of 6 items, each rated on a 5-point response scale: ranging from 1 (completely inconsistent) to 5 (completely consistent). The Cronbach’s alpha coefficient for the SASS-CS was 0.815.

### Sense of place scale

The Sense of Place Scale developed by Jorgensen and Stedman (60), was applied in this study to assess participants’ connection to their environment. The scale consists of 12 items, with certain wording revised to cater to the communication styles and real-life contexts of college students. It encompasses three dimensions: place identity (e.g., “The school and community where I live are relevant and reflect my sense of self”), place attachment (e.g., “The school and community I live in are relaxing and joyful”), place dependence (e.g., “When engaging in activities I value most, no other place can substitute the school and community where I reside”). Participants rated their agreement on a 5-point scale (1 = completely disagree to 5 = completely agree). The scale demonstrated acceptable internal consistency, with a Cronbach’s alpha coefficient of 0.743.

### Design

This quantitative study aimed to identify students’ resilience levels and four potential predictors using a questionnaire-based survey. Data mining method was chosen to process the collected data as it helps to predict psychological capital (67). In this paper, we chose the decision tree model to predict psychological resilience. A decision tree, as a non-linear discrimination method, is considered one of the most popular approaches for representing classifiers in statistics, machine learning, and data mining (68, 69). It classifies data by recursively splitting it into subsets based on the feature that best separates different outcomes. At each step, the algorithm selects the variable that maximizes the distinction between categories, creating branches until all subsets contain data from a single category. This stepwise process allows for intuitive and interpretable classification of complex datasets (70).

The decision tree model is based on the following considerations in this study: (i) the decision tree model produces intuitive and interpretable classification rules by training samples, classifying the new instances and outputting an easy-to-understand and top-down diagram. The diagram is a tree-like structure that consists of a root node, internal nodes, and leaf nodes, where internal nodes represent attribute tests, branches denote test outcomes, and leaf nodes indicate classification results, enabling rule inference at each node (71). (ii) decision tree algorithms maintain predictive accuracy despite multicollinearity and effectively handle complex predictor relationships. As a versatile machine learning and data mining method, decision trees are used for both classification and regression tasks, with categorical decision trees applied to categorical predictors and regression decision trees suited for continuous predictors (72). In this study, we focused on classifying students’ psychological resilience levels (high or low). In consequence, we built a predictive model using a classified decision tree algorithm and analyzed each predictor’s contribution to psychological resilience.

### Data coding

The sample was divided into: high and low psychological resilience groups, with 60% as the demarcation point. The choice to dichotomize psychological resilience was selected based on prior studies involving similar populations to ensure meaningful group comparisons, and this cutoff has been accepted in previous research (e.g., 70, 73). Five predictive factors were converted into binary variables based on consistent criteria (see **Table 1**).

**Table 1:** Summary statistics for variable codes and descriptive statistics.

Variable	Statistics for variable codes			Descriptive statistics			
	Coding	Number	Proportion	Full score	Mean value	Standard deviation	60% of the full score
School climate	0=low	194	32.00%	5	3.74	0.514	3
	1=high	413	68.00%				
Sense of place	0=low	239	39.00%	5	3.51	0.768	3
	1=high	368	61.00%				
Psychological resilience	0=low	251	41.00%	5	3.62	0.730	3
	1=high	356	59.00%				
Social anxiety	0=low	250	41.00%	5	3.51	0.837	3
	1=high	357	59.00%				
Creativity	0=low	295	49.00%	5	3.32	0.498	3
	1=high	312	51.00%				

### Decision tree model

To provide a complete theoretical reference, the underlying mechanics of the decision tree are described using information entropy and information gain ratio (71, 74).

The optimal branching variable and segmentation threshold of a decision tree can be determined by analyzing the decreasing rate of information entropy. Information entropy quantifies the impurity within a dataset and is described, based on Mitchell (71), as Equation 1:


(1) $$Entropy\left(D\right)=-\sum_{k=1}^{m}P_{k}log_{2}P_{k}$$

Here, D represents the training dataset with sample size m, and P_k_ denotes the probability of each category within the dataset. To assess the difference in information entropy of different classification methods, the information gain ratio is used. When the variable C is chosen to categorize the dataset D into n subsets, the information gain ratio is expressed as Equation 2 (74):


(2) $$Gain\;ration\left(D,C\right)=\frac{Entropy\left(D\right)-Entropy(D|C)}{Entropy\left(C\right)}$$

The algorithm in C5.0 utilizes the attribute with the highest information gain ratio as the splitting point, generates multiple subsets, and then creates multiple branches based on the attribute values. This process of selection continues until all subsets contain data belonging to a single category, thereby accomplishing inductive classification (68).

### Trimming of the decision tree

The post-pruning method was applied to trim the leaf nodes layer by layer. After building the decision tree, the dataset was recursed to each leaf node, and the mean square error (MSE) was calculated before and after pruning. A node was truncated if pruning resulted in a reduced MSE; otherwise, it was retained (70).

### Evaluation of the decision tree

70% of the sample data (n=423) was selected as training data and the remaining 30% (n=184) was selected as test data. Accuracy, precision and recall were requested as key metrics for the evaluation (69). The percentage of correctly classified samples out of the total number of samples was considered as accuracy. The proportion of positive samples correctly identified out of all predicted positive samples was considered as precision. The proportion of actual positive samples correctly identified by the model is considered recall.

### Data analysis

We used SPSS 22.0 for descriptive statistical analysis and Modeler 18.0 for decision tree modeling. Summary statistics was mainly applied to analyze the frequency distributions and central tendency of students’ psychological resilience and its predictive variables. The decision tree analysis was conducted with C5.0 algorithm that is an enhanced version of ID3 algorithm and C4.5 algorithm proposed by Quinlan (75, 76. The C5.0 decision tree algorithm is an advanced machine learning technique known for its high accuracy and ability to generate interpretable rule sets (77). The C5.0 algorithm is suitable for big data, and has faster running speed and superior predicting performance. Compared to its predecessors, the C4.5 algorithm significantly improved classification accuracy and reduced model-building time in big data analyses (78). The model was developed and validated following established best practices for predictive modeling (77).

## Results

### Descriptive statistics

**Table 1** presents the summary statistics. The mean value of psychological resilience was 3.74 with a standard deviation of 0.514, exceeding 60% of the maximum, which indicates that the majority of students exhibited a strong state of psychological resilience. To facilitate analysis, each variable was encoded: cases scoring above 60% of the full score were assigned a value of 1, while the remaining cases were coded as 0.

### Model predicting psychological resilience

**Figure 1:**
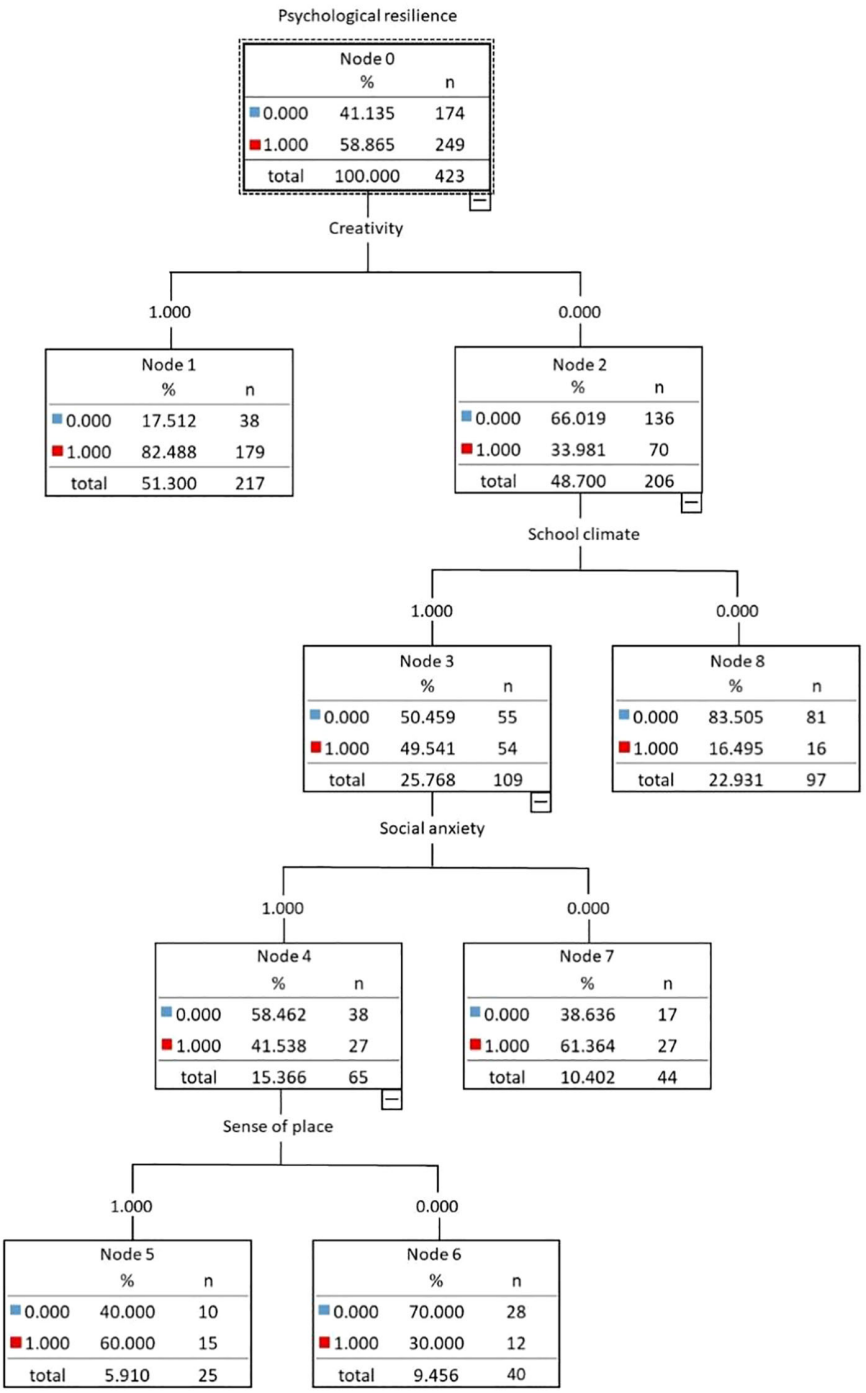
Prediction model for psychological resilience. The rectangle represents a node in a decision tree. The numerical values inside the node indicate the quantity and distribution of samples. The blue and red squares represent the proportion and volume of samples within the node, respectively. The value “n” represents the total number of samples in the node. The percentage value indicates the proportion of samples in the node relative to the total sample size. The “total” label is the cumulative number of samples in the node.

The decision tree model predicting psychological resilience is illustrated in **Figure 1**, with the associated prediction rules outlined below. Creativity emerges as the primary predictor of psychological resilience, indicating that students with stronger innovation ability are speculated to own higher psychological resilience with an accuracy rate of 82.49%. The resilience of less creative individuals depends on the recognition of school environment which is the second variable for psychological resilience prediction. Those students insensitive to school climate are evaluated to underperform in psychological self-adjustment with an accuracy rate of 83.51%. Nevertheless, for individuals who are easier to percept school atmosphere, social anxiety should be further considered. When students feel less social anxiety, they more equipped to manage difficulties and keep a good mental state (61.36%). Otherwise, they need to have a strong sense of place, so as to prompt the formation of high psychological resilience (60%).

**Figure 2:**
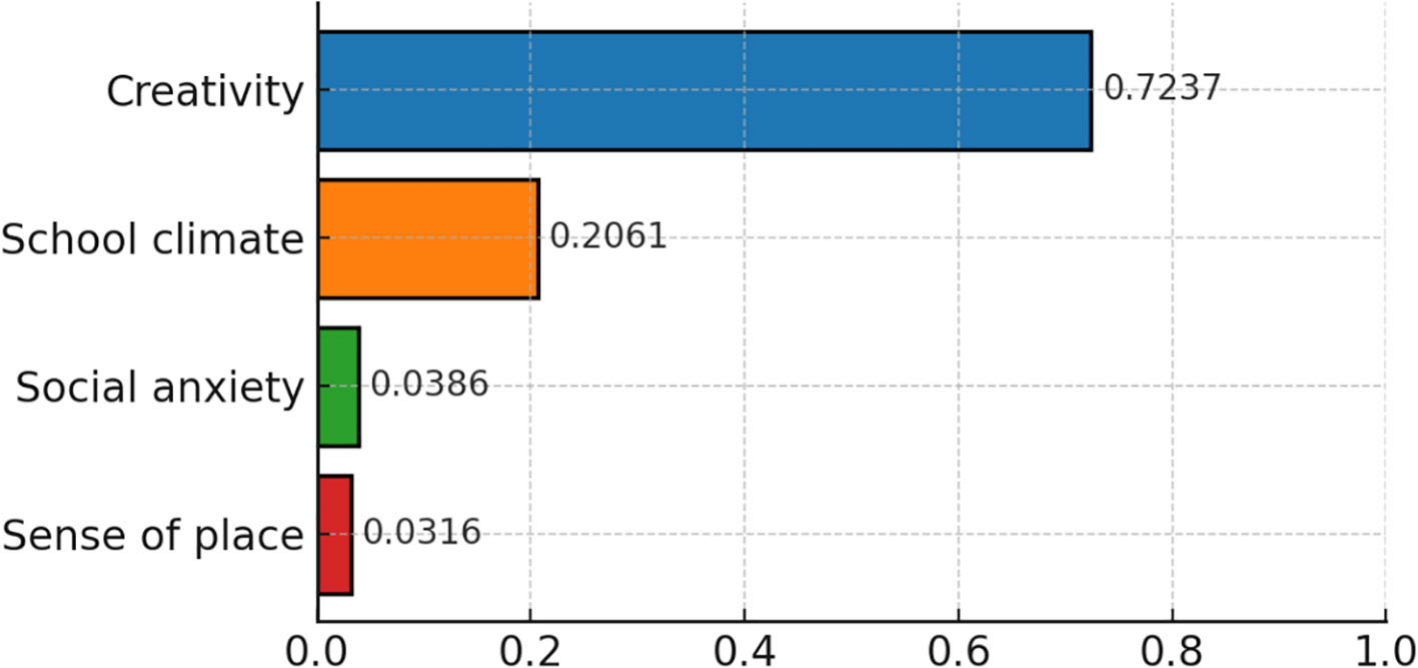
The predictor variables, in order of importance.

**Figure 2** illustrates the importance of each variable in the predictive model which reflects the contribution to the overall prediction. Among the four predictors, creativity is the most important variable to predict psychological resilience. School climate ranks second in order of significance and its prediction power is less than one third of creativity. Social anxiety and sense of place are the third and fourth important predictors respectively which are of equal importance and both weak in prediction.

### The evaluation of the prediction model

The confusion matrix and classification accuracy are presented respectively in **Table 2** and **Table 3**. The model accuracy for the testing data set is 78.26%. On the basis of computation rule of precision and recall, the model’s precision with the testing data set is 77.23% and the model recall of the testing data set is 88.79%. In this classification, the AUC value of the training data set is 0.801 and the AUC of the testing data set is 0.821, which is directly obtained from the model evaluation report. According to Fawcett (79), the AUC value of our model for predicting psychological resilience is greater than 0.5, indicating that the model is more accurate than a random guessing. The corresponding Gini coefficient was 0.601 for training data and 0.642 for testing data. Furthermore, the F1-Score is about 82.6% in our model, which can be calculated as F1-score = 2 × (Precision × Recall)/(Precision + Recall).

**Table 2:** Confusion matrix.

Data category	Predicted level	Predicted class	
		low	high
Actual class of training data	low	109 (True Negatives)	65 (False Positives)
	high	28 (False Negatives)	221 (True Positives)
Actual class of testing data	low	49 (True Negatives)	28 (False Positives)
	high	12 (False Negatives)	95 (True Positives)

* Accuracy refers to the ratio of correctly classified cases compared to the total number of samples. Thus, accuracy = (49 + 95)/(49 + 28 + 12 + 95) = 0.783.

* Precision represents the proportion of positive prediction cases that are real positive samples. Thus, precision = 95/(28 + 95) = 0.772.

* Recall (also known as Sensitivity) is defined as the proportion of true positive cases in the sample that are correctly identified. Thus, recall = 95/(12 + 95) = 0.888.

* Specificity measures the proportion of actual negative cases (low-resilience students) that are correctly identified by the model. Thus, Specificity = True Negatives/(True Negatives + False Positives) = 49/(49 + 28) = 0.636.

* Balanced Accuracy is the arithmetic mean of Sensitivity and Specificity, providing a robust metric for evaluating performance on imbalanced datasets. Thus, Balanced Accuracy = (Sensitivity + Specificity)/2 = [95/(12 + 95) + 49/(49 + 28)]/2 = 0.762.

**Table 3:** Classification accuracy.

Data category	Predicted level	Number	Proportion
Training data	Correct	330	78.01%
	Wrong	93	21.99%
	Total	423	
Testing data	Correct	144	78.26%
	Wrong	40	21.74%
	Total	184	

## Discussions

This article constructed a four-factor model using the C5.0 decision tree algorithm to predict the psychological resilience, and figured out the predictive strength of four factors. Both accuracy and precision values approach to 80%, while the recall value is nearly 90%, verifying the reliability of the predicting outcomes.

According to data analysis in **Figure 2**, creativity emerged as the strongest predictor of psychological resilience among four factors in our model. Triangular theory of creativity proposed by Sternberg described that creative individual defy the general public, their own limitations and/or prevailing zeitgeist through various forms of defiance (80, 81). In fact, this process parallels the confrontation with self and environment when people recovery and bounce back from adversity and enhance coping skills. Consequently, we can infer that the built-up process of creativity is partially overlapped with exhibition of mental resilience, a connection that appears salient in our sample. Moreover, (82) maintained that high levels of creativity are often associated with activation of positive mood that generally contributes to greater resilience. McFadden and Basting (83) argued that creative involvement, as a manifestation of and a bolster to resilience, can reinforce it on biological, psychological, and social levels. In summary, creativity is related to the social aspect of psychological resilience, and participation in creative activities and psychological resilience are mutually reinforcing (32, 83, 84). For college students, this suggests that innovative behaviors are associated with their problem-solving ability, enrich their sense of achievement, and are linked to higher level of resilience. Notably, it is logical to conclude that creativity should be considered a key predictor for psychological resilience in this specific population of Chinese college students, suggesting that fostering innovative behaviors may support problem-solving ability and enrich the sense of achievement, thereby enhancing resilience. Moreover, it is crucial to interpret these findings within their specific context. The predictive strength of these factors, particularly creativity, was identified within a sample of Chinese normal university students. Therefore, the conclusions should be qualified as context-dependent and may reflect the unique characteristics of this population.

As the second most influential factor, school climate, despite its predictive power triple lower than creativity, is associated with the reinforcement of psychological resilience through its three subordinates: teacher support, peer support and opportunities for autonomy in the classroom. As one kind of social support, teacher support (e.g., getting along well with teacher, receiving care and support from teacher) enables students to feel in a warm and friendly environment that reduces the impact of negative emotions on physical and mental well-being (85) and fosters self-recognition and favorable self-assessment (86, 87). Positive emotions assist in behavioral and emotional regulation that support the development of inner resources (e.g., problem solving, self-efficacy), finally result in the enhancement of psychological resilience (86, 88). Positive peer relationships are critical for the psychological wellbeing of students and viewed as exterior energy that boost resilience (56, 89). When positive feelings and beliefs about peers exist, youth may develop resilience (39) and obtain resilience-driven outcomes (90). One of the major needs of successful youth development is nourishing and growth-enhancing opportunity provided by teachers and staff in school, associated in some important ways with students’ academic performance and mental wellness (91). Jia et al. (37) confirmed that classroom autonomy opportunities showed a strong positive association with self-esteem and a negative association with depressive symptoms. Specifically, students’ embeddedness in autonomous activities such as rule-making, study schedule arranging and course-selecting would facilitate a feeling of self-identity (92) and obtain the relationship resource to cope resiliently and constructively with classroom challenges (93). Overall, students’ perception of school climate can serve as a valuable indicator of their level of psychological resilience.

Social anxiety and sense of place are in nearly equal forecasting ability towards students’ resilience, the significance of which apparently fell behind creativity and school climate. Unlike other predictive factors in this model, social anxiety was found negatively correlated to psychological resilience (49, 53). People suffering from social anxiety disorder have cognitive errors including low self-assessing of ability, over-estimated risk, catastrophizing symptoms and lack of social skills that would create a barrier in initiating or sustaining social relationships and receive unfavorable response from the external world (94, 95), which is exactly opposed to the need for psychological resilience. Social anxiety disorder impairs normal functioning in academic, workplace, or daily life settings (50), leading individuals to negatively evaluate themselves and avoid social interactions due to a fear of failure (51, 96). Ma (52) also maintained that psychological health of students especially motivation and adaptability may be severely affected by social anxiety. Those symptoms caused by social anxiety are adverse for students to accommodate themselves to changes, endure negative events and keep high self-identity, corresponding to lower resilience. Therefore, social anxiety can be used to predict resilience from the opposite direction.

Although sense of place is the least important predictive factor, to some extent it exerts a positive impact on enhancing psychological resilience. Hess et al. (97) proposed that place focus facilitate resilience as identity and sense of place are crucial to community resilience, public health, social relations and well-being (98). Burley et al. (99) inferred that residents’ resilience is derived from strong degree of place attachment by exploring how residents reacts to environmental change after disaster. Resilience here represents both physical stability of community and mental adjustment ability of individuals when confronted with challenges. Sense of place was considered as significant foundation of community resilience (100–102), of which place attachment may support adaption when residents meet with threatening changes (103). During the adapting process, individuals develop coping capacity and reconstruct meaning about place through self-reflection and value renewal, therefore enhance psychological resilience. Similarly, strong sense of place is associated with college students to involve in campus life as quickly, adapt to changes, and dynamically maintain physical and mental balance. Namely, in the process of achieving harmony between individuals and place, students’ psychological resilience would potentially be strengthened simultaneously. Therefore, sense of place could be viewed as a reference factor to forecast psychological resilience.

## Implications

The psychological resilience of college students mutually reinforcing with mental health has attracted great attention. Based on our findings, we propose some suggestions for teachers and college counselors to elevate students’ psychological condition. First, adequate attention and action should be put into the development of creativity that is linked to mental resilience. Specifically, engagement in artistic events, academic competition and innovation competition would facilitate students’ innovation while training programs and physical activities embedded in students’ daily life also play a catalytic role in creativity (104, 105). Second, teachers and college counselors should make students feel a sense of security and belonging through creating positive school atmosphere. In this circumstance, enough teacher support should be offered in everyday lives or when students seek help, peer bonds need to be persistently restored or strengthened and students’ autonomy and independence in classroom should be fully supported and encouraged. Third, more care and understanding are needed for students with social phobia who might be guided out of virtual world and enclosure space via ice-breaking activities. Last, the establishment of sense of place is rooted in every link that always takes a long time. Teachers and college counselors need to infiltrate and cultivate students’ attachment to the campus in every detail. In short, excessively immersed in the virtual world of mobile phones is correlated with impaired social ability and sense of place, negative emotions brought by which may overshadow creative processes and resilience level.

## Limitations and future directions

Three limitations should be noted in this study. First, this research is based on a cross-sectional design with data collected at a single time point. Consequently, the predictive model we developed is theoretical and demonstrates associative relationships rather than causal effects. The findings indicate which factors are strongly related to resilience within our model, but the design does not permit causal inferences to be drawn regarding the influence of these predictors on resilience over time. Second, our sample (n =607) was randomly selected in a certain university, which can be expanded to schools in different regions and levels. To enhance the generalizability of the findings, future research should expand the sample to include students from diverse geographical regions and institutional types. Third, the factors influencing psychological resilience contained in this research are limited. Psychological factors such as deliberate rumination and posttraumatic growth are markedly related to resilience and their predictive ability have not been explored. As such, our prediction model needs further refinement while this study provides a new path for the prediction of psychological resilience.

## Conclusions

This article established a model with four factors predicting psychological resilience based on the decision tree with the C5.0 algorithm. Our model with an accuracy rate of 78.26% demonstrated good forecasting ability. The predictive power of the four factors was ranked in order of importance: creativity, school climate, social anxiety and sense of place. Creativity and school climate had obvious superiority in predicting resilience, and social anxiety and sense of place played a similar and weak role. Although our prediction model needs further improvement on research method, sample size and predictor range, it does provide reference value for guiding the work of teachers and college counselors in understanding the factors associated with the psychological level of college students.

## Data Availability

The original contributions presented in the study are included in the article/supplementary material. Further inquiries can be directed to the corresponding author.
